# A benzimidazole derivative exhibiting antitumor activity blocks EGFR and HER2 activity and upregulates DR5 in breast cancer cells

**DOI:** 10.1038/cddis.2015.25

**Published:** 2015-03-12

**Authors:** B Chu, F Liu, L Li, C Ding, K Chen, Q Sun, Z Shen, Y Tan, C Tan, Y Jiang

**Affiliations:** 1 Department of Chemistry, Tsinghua University, Beijing 100084, People's Republic of China; 2 The Ministry-Province Jointly Constructed Base for State Key Lab-Shenzhen Key Laboratory of Chemical Biology, Graduate School at Shenzhen, Tsinghua University, Shenzhen 518055, People's Republic of China; 3 Shenzhen Anti-Tumor Drug Development Engineering Laboratory, Graduate School at Shenzhen, Tsinghua University, Shenzhen 518055, People's Republic of China; 4 Shenzhen Kivita Innovative Drug Discovery Institute, Shenzhen 518055, People's Republic of China; 5 Department of Pharmacology and Pharmaceutical Sciences, School of Medicine, Tsinghua University, Beijing 100084, People's Republic of China

## Abstract

Aberrant expression or function of epidermal growth factor receptor (EGFR) or the closely related human epidermal growth factor receptor 2 (HER2) can promote cell proliferation and survival, thereby contributing to tumorigenesis. Specific antibodies and low-molecular-weight tyrosine kinase inhibitors of both proteins are currently in clinical trials for cancer treatment. Benzimidazole derivatives possess diverse biological activities, including antitumor activity. However, the anticancer mechanism of 5a (a 2-aryl benzimidazole compound; 2-chloro-*N*-(2-*p*-tolyl-1*H*-benzo[*d*]imidazol-5-yl)acetamide, C_16_H_14_ClN_3_O, MW299), a novel 2-aryl benzimidazole derivative, toward breast cancer is largely unknown. Here, we demonstrate that 5a potently inhibited both EGFR and HER2 activity by reducing EGFR and HER2 tyrosine phosphorylation and preventing downstream activation of PI3K/Akt and MEK/Erk pathways *in vitro* and *in vivo*. We also show that 5a inhibited the phosphorylation of FOXO and promoted FOXO translocation from the cytoplasm into the nucleus, resulting in the G1-phase cell cycle arrest and apoptosis. Moreover, 5a potently induced apoptosis via the c-Jun N-terminal kinase (JNK)-mediated death receptor 5 upregulation in breast cancer cells. The antitumor activity of 5a was consistent with additional results demonstrating that 5a significantly reduced tumor volume in nude mice *in vivo*. Analysis of the primary breast cancer cell lines with HER2 overexpression further confirmed that 5a significantly inhibited Akt Ser473 and Bad Ser136 phosphorylation and reduced cyclin D3 expression. On the basis of our findings, further development of this 2-aryl benzimidazole derivative, a new class of multitarget anticancer agents, is warranted and represents a novel strategy for improving breast cancer treatment.

The ERBB family of transmembrane receptor tyrosine kinases (RTKs) includes four closely related members: epidermal growth factor receptor (EGFR) (ERBB1, HER1), human epidermal growth factor receptor 2 (HER2) (ERBB2, Neu), HER3 (ERBB3) and HER4 (ERBB4).^1^ Binding of ligands to the extracellular domain of EGFR, HER3 and HER4 induces the formation of receptor homodimers or heterodimers and autophosphorylation of the intracellular domain of the receptors.^2, 3^ HER2 does not bind any of the ERBB ligands directly, but it can heterodimerize with other ERBB family members.^4^ Active EGFR and HER2 induce transphosphorylation of ERBB and trigger intracellular signaling pathways involved in the proliferation response.^5^ Because aberrant ERBB signaling pathways correlate with human cancers, RTKs have been studied intensively in recent decades. It is known that overexpression of HER2 is found in about 20% of breast cancer patients, leading to aberrant signaling of the PI3K/Akt and MEK/Erk pathways, and is correlated with malignant transformation, chemotherapy resistance and poor prognosis.^1, 6, 7^ Meanwhile, aberrant EGFR activity was also observed during pathogenesis and progression of lung and breast cancers.^8, 9^ Therefore, a promising approach may lie in the development of chemotherapeutic strategies exploiting the deregulation of target ERBB to create cancer treatments with both preventive and therapeutic potential. Clinically, small-molecule competitive tyrosine kinase inhibitors, which compete with ATP in the receptor kinase domain, have been used to block EGFR or HER2 intracellular tyrosine kinase activity.^10^ Alternative treatments using anti-EGFR or anti-HER2 antibodies, which bind to the extracellular domain of ERBB, have been used to prevent ligand binding, receptor activation and/or induce receptor internalization.^11, 12^ Lapatinib, a selective small-molecule inhibitor of EGFR and HER2 tyrosine kinases, quickly disables EGFR and HER2 signaling, resulting in the inhibition of the PI3K/Akt and MEK/Erk pathways,^13^ subsequently inducing proliferation arrest and apoptosis in EGFR- and HER2-dependent cancer cell lines.

Apoptosis is activated in response to proapoptotic stimuli via two distinct signaling pathways: the extrinsic (or death receptor (DR)) pathway and intrinsic (or mitochondrial) pathway.^14^ The extrinsic pathway is triggered by members of the tumor necrosis factor (TNF) superfamily, which bind and activate their corresponding DRs. For example, binding of TNF-related apoptosis-inducing ligand (TRAIL) to the extracellular domains of the DR4 and DR5 promotes clustering of these receptors, and then induces apoptosis. The TRAIL receptors DR4 and DR5 are important proapoptotic molecules that belong to the TNF receptor superfamily.^15^ While binding to their ligand TRAIL, DR4 and DR5 transmit apoptotic signals through the rapid activation of caspase-8. By initiating the activation of caspase cascades, DR4 and DR5 directly induce apoptosis of target cells, preferentially in transformed or malignant cells.^15, 16^ Bioymifi, a small-molecule compound, directly targets DR5 to induce DR5 clustering and aggregation, leading to apoptosis in human cancer cells.^17^ Similarly, lapatinib induces DR5 upregulation through the activation of the c-Jun N-terminal kinase (JNK)/c-Jun signaling axis, leading to more efficient induction of apoptosis in colon cancer cells.^18^ These studies suggest that upregulation of DR4 and/or DR5 has an important role in apoptosis of various cancer cell types *in vitro* and *in vivo*.^17, 18, 19^ Therefore, agents that induce upregulation of DR4 and/or DR5 may have the potential for the clinical management of cancer.

Multiple studies have demonstrated various bioactivities of benzimidazole derivatives, including anti-inflammatory,^20^ antioxidant,^21^ antiviral,^22^ antimicrobial^23^ and anticarcinogenic activity.^24, 25, 26, 27, 28^ Their antitumor activity may act through the inhibition of poly (ADP-ribose) polymerase-1 (PARP-1),^24^ topoisomerase I,^25^ cell cycle checkpoint kinase 2^26^ and tyrosine kinases.^27, 28^ One of these analogs, 2-aryl benzimidazole compound (5a; 2-chloro-*N*-(2-p-tolyl-1*H*-benzo[d]imidazol-5-yl)acetamide, C_16_H_14_ClN_3_O, MW299) (Figure 1a), is a novel benzimidazole derivative, which was found to induce apoptosis in a human hepatocellular carcinoma cell (Hep G2),^27^ but the mechanism by which it induces apoptosis and antitumor activity in breast cancers is largely unknown. In this study, we demonstrate that 5a-induced cell cycle arrest and apoptosis by inhibiting EGFR and HER2 activity and downstream activation of PI3K/Akt and MEK/Erk pathways. 5A also induced apoptosis through JNK-mediated DR5 upregulation in human breast cancer cells. This study demonstrates that 5a is a novel multitarget antitumor drug candidate that has great potential as a novel agent for anticancer therapy.

## Results

### 5A exhibits cytotoxicity and apoptotic activity toward human breast cancer cells

The anticancer effects of 5a on human breast cancer cells were evaluated *in vitro* using a panel of nine established breast cancer cell lines. These cell lines expressed widely varying levels of EGFR and HER2, including EGFR-positive, HER2-positive and EGFR/HER2-double-negative cells (Supplementary Figure S1a). As shown in Table 1, 5a exhibited broad-spectrum inhibition of breast cancer cell growth, with IC_50_ values of 2–9 *μ*M by an MTT (3-(4,5-dimethylthiazol-2-yl)-2,5 diphenyl tetrazolium bromide) assay. From the MTT results, we found that human breast cancer cell lines containing *EGFR* gene amplification with high expression of EGFR (MDA-MB-468) showed an IC_50_ value of 3.31 *μ*M, whereas the other cell lines with *EGFR* gene amplification but with lower EGFR expression, such as HCC1937, showed an IC_50_ of 9.02 *μ*M. The breast cancer cells that overexpress HER2 exhibited a similar response to 5a. The IC_50_ value from a cell line with high expression of HER2 (BT-474) was 3.58 *μ*M, whereas the other cell lines with lower levels of HER2 expression, such as MDA-MB-453, showed an IC_50_ equal to 4.91 *μ*M. These results suggest that *EGFR* and *HER2* gene amplification and their proteins overexpression are consistent with the higher sensitivity to 5a *in vitro* across various tested cell lines. According to these results, we propose that the antitumor activity of 5a in breast cancer cells may result from inhibition of EGFR and HER2 activity. However, we also found that breast cancer cell lines with lower EGFR and HER2 expression (ZR-75-1 and MCF-7) showed low IC_50_, from 1.81 *μ*M to 2.99 *μ*M. These results indicate that 5a not only targets EGFR and HER2 but may also target other gene(s) and/or protein(s) in breast cancer cells. To determine cytotoxicity, we treated a normal breast cell line, MCF-10 A, with 5a and found that the IC_50_ value of 5a against MCF-10 A was 17.33 *μ*M, suggesting that 5a had low cytotoxicity toward this normal breast cell line. The IC_50_ value of lapatinib against breast cancer cells was used as positive control.

**Figure 1:**
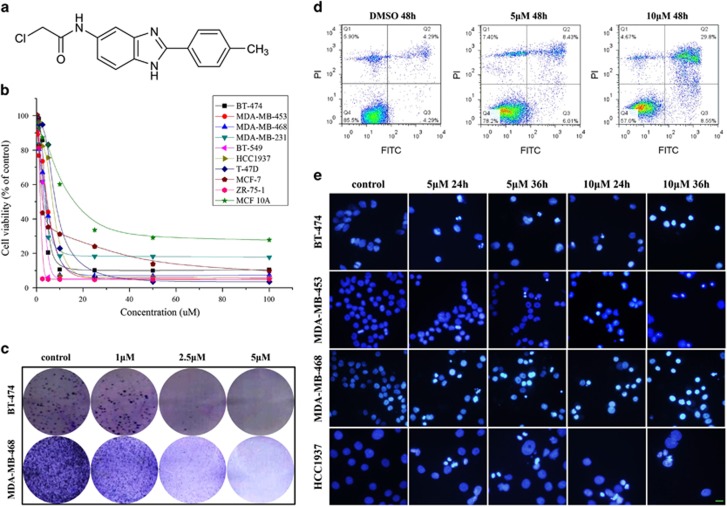
5A exerted cytotoxic activity in breast cancer cells. (**a**) The structure of 5a. (**b**) Breast cancer cells were treated with increasing concentrations of 5a for 72 h, cell viability was analyzed by MTT assay. (**c**) Cell viability, as determined by colony formation assay, was assessed in breast cancer cells treated with 5a. (**d**) Cells were cultured with 5  or 10 *μ*M 5a for 48 h and then subjected to apoptosis assay, using flow cytometry. (**e**) Breast cancer cells treated with 5 or 10 *μ*M 5a for indicated time points were stained with Hoechst 33258 dye; apoptotic bodies and chromatin condensation were revealed under a fluorescence microscopy; scale bar, 20 *μ*m

The effects of 5a on cell viability and its proapoptotic activity were assessed using breast cancer cells. The results show that treatment of 5a resulted in a dose-dependent reduction in the viability of breast cancer cells (Figure 1b). The number of colonies formed from cells cultured with 5a was significantly lower, with much smaller colony size than in the control group (Figure 1c and Supplementary Figure S1b). Next, we characterized the proapoptotic activity of 5a by flow cytometry, treatment with 5a at 10 *μ*M showed induction of 38.35% cells to apoptosis in MDA-MB-453 cells (Figure 1d). Nuclear staining with Hoechst 33258 showed that 5a treatment resulted in condensed chromatin and apoptotic bodies, which are morphological hallmarks of apoptotic cell death (Figure 1e). These data, together with the MTT results, provide strong evidence that 5a exhibited antitumor properties by inducing apoptosis and reducing both proliferation and viability of breast cancer cells.

### 5A inhibited EGFR and HER2 tyrosine phosphorylation and downstream activation of PI3K/Akt and MEK/Erk pathways *in vitro*

**Figure 2:**
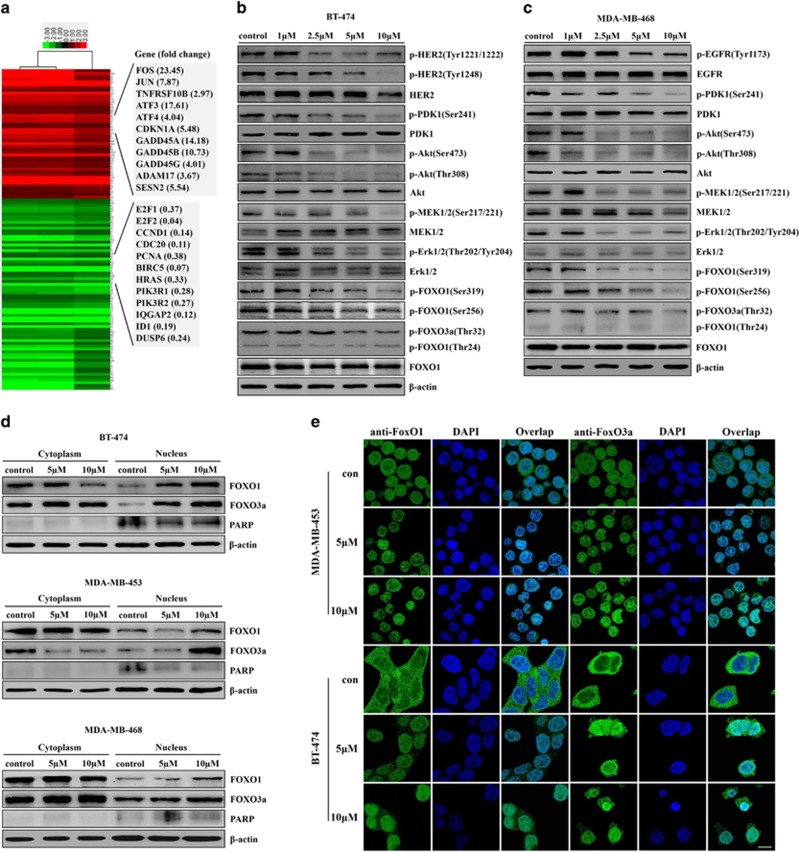
5A inhibited EGFR/HER2 tyrosine phosphorylation and downstream signaling pathways. (**a**) Hierarchical clustering and relative gene expression differences in 5a-induced MDA-MB-453 cells relative to the control cells. Signal intensity of each gene from the microarray was compared with control cells (fold change ≥2). Microarray analysis was performed in triplicate for each condition. (**b** and **c**) BT-474 and MDA-MB-468 cells were treated with 5a for 8 and 12 h, respectively, with different concentrations as indicated; cell lysates were analyzed by immunoblotting with the antibodies indicated. (**d**) BT-474, MDA-MB-453 and MDA-MB-468 cells were treated with 5a for 12 h with different concentrations as indicated; fractionation of nuclear and cytoplasmic proteins were performed and analyzed by western blot. (**e**) BT-474 and MDA-MB-453 cells were treated with 5a for 12 h with different concentrations as indicated, and then subjected to immunofluorescence analysis; scale bar, 20 *μ*m

Using microarray gene expression analyses, we discovered that 5a had a significant impact on the cell cycle and MEK/Erk signaling pathways, and their *P*-values were 5.62 × 10^−21^ and 2.04 × 10^−17^, respectively. These results suggest that 5a might exert its antitumor activity through the MEK/Erk signaling pathway, which is a downstream pathway of both EGFR and HER2. We also searched for genes that exhibited the greatest changes in expression in the 5a-induced MDA-MB-453 cells relative to the control cells (Figure 2a). Several of these genes, including *FOS*, *JUN*, *TNFRSF10B* (DR5), *CDKN1A* (p21), *E2F1*, *E2F2* and *CDC20*, have been reported to be involved in apoptosis and cell cycle arrest. Therefore, these results suggest that 5a could specifically target EGFR and HER2, and the antitumor activity of 5a may act through the inhibition of EGFR and HER2 activity or other gene and/or protein (such as DR5) to induce cell cycle arrest and apoptosis in breast cancer cells.

The effects of 5a on the activation of HER2, as well as downstream proliferation and survival pathways, were examined in BT-474 and MDA-MB-453 cells, which express high levels of phosphorylated HER2. 5A treatment lead to an obvious decrease in phosphorylated HER2 in a time- and dose-dependent manner in these two cell lines (Figure 2b and Supplementary Figures S2a and c). A similar time- and dose-dependent relationship was observed in MDA-MB-468 and HCC1937 cells, which overexpress EGFR (Figure 2c and Supplementary Figures S2b and d). As EGFR and HER2 overexpression is associated with the activation of downstream PI3K/Akt and MEK/Erk pathways, we assessed the effect of 5a on these pathways in breast cancer cells. It was found that 5a could inhibit the phosphorylation of PDK1, Akt, MEK1/2 and Erk1/2, while the total steady state of these proteins remained unchanged (Figures 2b and c and Supplementary Figure S2). 5A was also found to inhibit effectively the phosphorylation of the Akt and Erk1/2 substrate, FOXO (Figures 2b and c and Supplementary Figure S2). When FOXO is dephosphorylated, it translocates from the cytoplasm into the nucleus, where it regulates diverse arrays of transcriptional targets to promote cell cycle arrest and apoptosis. Western blot and immunofluorescence analyses confirm that 5a significantly induced FOXO translocation from the cytoplasm into the nucleus in breast cancer cells (Figures 2d and e). These results suggest that EGFR and HER2 and their downstream PI3K/Akt and MEK/Erk pathways could be the targets of 5a in breast cancer cells.

### 5A induced G1 arrest and apoptosis in breast cancer cells by inhibiting EGFR and HER2 activity

Activated EGFR and HER2 are known to specifically phosphorylate targeted proteins at serine and threonine sites and subsequently regulate diverse cellular activities, such as gene expression, mitosis, cell cycle and apoptosis through the PI3K/Akt and MEK/Erk pathways. In this study, we examined the involvement of EGFR and HER2 in 5a-induced cell cycle arrest and apoptosis.

**Figure 3:**
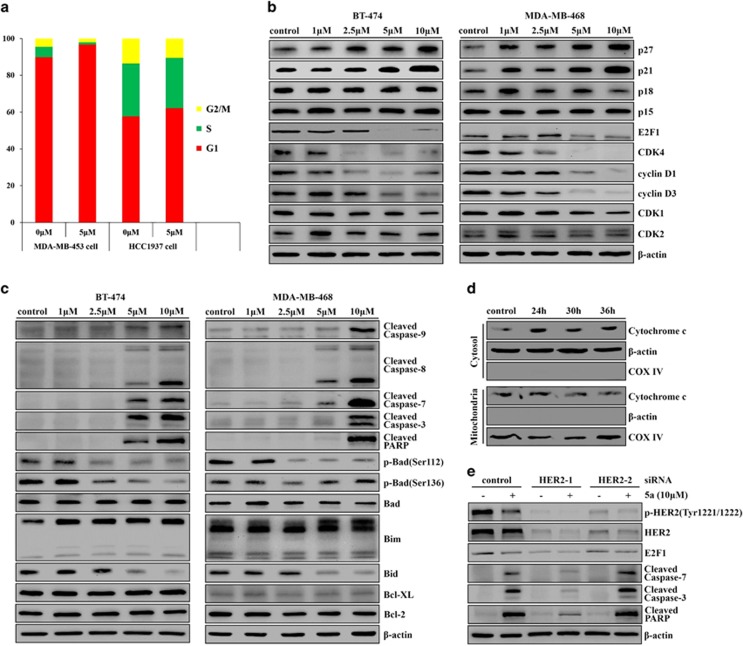
5A induced G1 arrest and apoptosis by inhibiting EGFR and HER2 activity. (**a**) MDA-MB-453 and HCC1937 cells were treated with 5 *μ*M 5a or dimethylsulfoxide (DMSO) for 24 h. The fractions of cells in G1, S and G2/M were determined by flow cytometry. (**b**) BT-474 and MDA-MB-468 cells were treated with 5a for 8 h and 12 h, respectively, with different concentrations as indicated; cell lysates were analyzed by immunoblotting with the antibodies indicated. (**c**) BT-474 and MDA-MB-468 cells were treated with 5a for 16 h and 12 h, respectively, with different concentrations as indicated; cell lysates were analyzed by immunoblotting with the antibodies indicated. (**d**) MDA-MB-453 cells were treated with 10 *μ*M 5a for 24, 30 or 36 h. Cytosolic and mitochondrial fractions were isolated to examine the distribution of cytochrome *c*. *β*-Actin and COX-IV (cytochrome *c* oxidase subunit 4) were used as the cytosolic and mitochondrial markers, respectively. (**e**) BT-474 cells were transfected with control or HER2 siRNA for 48 h and then treated with 10 *μ*M 5a for 14 h, cell lysates were analyzed by immunoblotting with the antibodies indicated

Following 5a treatment, two types of breast cancer cells (HER2-positive and EGFR-positive) were noticeably arrested in the G1 phase of the cell cycle, with a concomitant loss of the S- and G2/M-phase cell populations (Figure 3a). The effects of 5a on protein expression of INK4 (p15 and p18) and CIP/KIP (p21 and p27) families of cyclin-dependent kinase (CDK) inhibitors were studied, as they are negative regulators of the G1/S-phase progression. Interestingly, it was demonstrated that 5a could significantly upregulate protein expression of p27 and p21, which control the cell cycle progression at G1, but could not alter the expression of p18 and p15 in breast cancer cells. Furthermore, E2F1, CDK4 and cyclin D, which are also important for cell cycle progression, decreased in response to 5a treatment. However, 5a showed no obvious effect on the protein levels of CDK1 and CDK2 (Figure 3b and Supplementary Figure S3). Therefore, the downregulation of E2F1, CDK4, cyclin D1 and cyclin D3 and upregulation of p27 and p21 in breast cancer cells likely contributed to the G1 cell cycle arrest induced by 5a.

The possible mechanism of 5a-induced apoptosis was also examined in breast cancer cells. We found that 5a increased the active (cleaved) caspase-9 and caspase-8 levels in a time- and concentration-dependent manner. The subsequent activation of caspase-7, caspase-3 and PARP were also detected (Figure 3c and Supplementary Figure S4). It has been reported that Bcl-2 family proteins are involved in caspase-dependent apoptosis. Results showed that 5a noticeably induced Bim expression, but reduced the levels of p-Bad (Ser112) and p-Bad (Ser136). However, 5a treatment did not affect the levels of Bcl-XL and Bcl-2. Bid, a known substrate of caspase-8, was cleaved, as indicated by the decreases in its expression levels during 5a treatment (Figure 3c and Supplementary Figure S4). Western blot analysis also demonstrated that cytochrome *c* was released from mitochondria to the cytoplasm in 5a-exposed cells (Figure 3d). These data suggest that 5a-induced apoptosis, through both extrinsic and intrinsic pathways, and the intrinsic pathway could be mitochondrial-dependent.

Furthermore, HER2 was knocked down using two different small interfering RNA (siRNA) oligos in BT-474 cells to detect whether HER2 was required for 5a-induced cell cycle arrest and apoptosis. In BT-474 cells, siRNA oligos induced HER2 downregulation (Supplementary Figure S5a) and obvious suppression of activities of HER2 (Figure 3e). Notably, depletion of HER2 rescued the breast cancer cells from 5a-induced E2F1 downregulation and also abrogated the effect of 5a on the activation of caspase-7, caspase-3 and PARP (Figure 3e), suggesting that HER2 is the predominant target for 5a-induced cell cycle arrest and apoptosis. In conclusion, these results suggest that HER2 was involved G1 arrest and apoptosis induced by 5a in breast cancer cells.

### 5A induced DR5 upregulation through activation of JNK signaling

**Figure 4:**
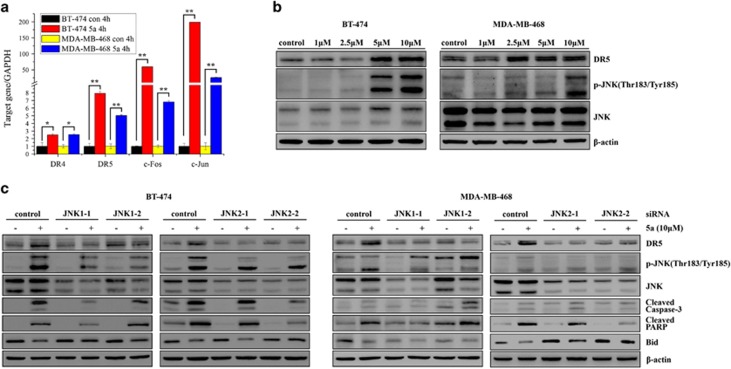
5A induced activation of DR5 through the JNK signaling pathway. (**a**) BT-474 and MDA-MB-468 cells were treated with 5a at 10 *μ*M for 4 h; real-time PCR results for gene expression of indicated genes in these cell lines are shown relative to control cells (*P<0.05; **P<0.001). (**b**) BT-474 and MDA-MB-468 cells were treated with 5a for 12 h with different concentrations as indicated; cell lysates were analyzed by immunoblotting with the antibodies indicated. (**c**) BT-474 and MDA-MB-468 cells were transfected with control or JNK siRNA for 48 h and then treated with 10 *μ*M 5a for 12 and 8 h, respectively; cell lysates were analyzed by immunoblotting with the antibodies indicated

As the proapoptotic response induced by 5a was associated with caspase-9 and caspase-8 cleavage, and cleaved caspase-8 is an initiator caspase for extrinsic DR signaling, we speculated that 5a induces apoptosis not only through intrinsic apoptosis pathways but also through DRs, such as DR4 and DR5,^29, 30^ mediated extrinsic apoptosis pathways. As microarray gene expression analyses revealed, 5a had a significant impact on DR5 expression, and this gene was overexpressed 2.97-fold in 5a-induced MDA-MB-453 cells relative to the control (Figure 2a). Real-time PCR analysis also revealed that 5a increased DR5 and DR4 mRNA levels by 5- to 8-fold and 2.5-fold, respectively, in MDA-MB-468 and BT-474 cells, both of which have mutant p53^31, 32^ (Figure 4a). This suggests that DR5 may have a more important role than DR4 in mediating 5a-induced apoptosis. Therefore, we next examined the expression of DR5 at the protein level and found that DR5 protein expression increased after treatment with 5a in the two tested cell lines (Figure 4b and Supplementary Figure S5b). We also noted that c-Jun and c-Fos, two well-known JNK substrates, were generally upregulated by 5a in the breast cancer cell lines (Figure 2a). Analysis by real-time PCR further confirmed that 5a could significantly increase the expression of c-Jun and c-Fos (Figure 4a). After 5a treatment, phosphorylation of JNK was also detected (Figure 4b and Supplementary Figure S5b). Collectively, these results suggest that 5a might activate the JNK/c-Jun pathway to induce DR5 upregulation in human breast cancer cells.

To determine the implications of increased JNK phosphorylation in 5a-induced DR5 upregulation and apoptosis, we inhibited JNK1 and JNK2 expression with siRNA (Supplementary Figure S5c) and examined the effects of 5a on DR5 expression, caspase activation and apoptosis. As expected, si-JNK1-1 and si-JNK2-2 completely abolished not only 5a-induced increase in p-JNK but also blocked 5a-induced upregulation of DR5 (Figure 4c). Furthermore, 5a-induced activation of caspase-3, PARP and cleavage of Bid were abrogated by si-JNK1-1 and si-JNK2-2 (Figure 4c). Collectively, these results suggest that 5a could exert antitumor activity and induce DR5 upregulation through the activation of JNK signaling in breast cancer cells.

### 5A exerted an antitumor effect *in vivo* through the inhibition of HER2 tyrosine phosphorylation and downstream signaling components

**Figure 5:**
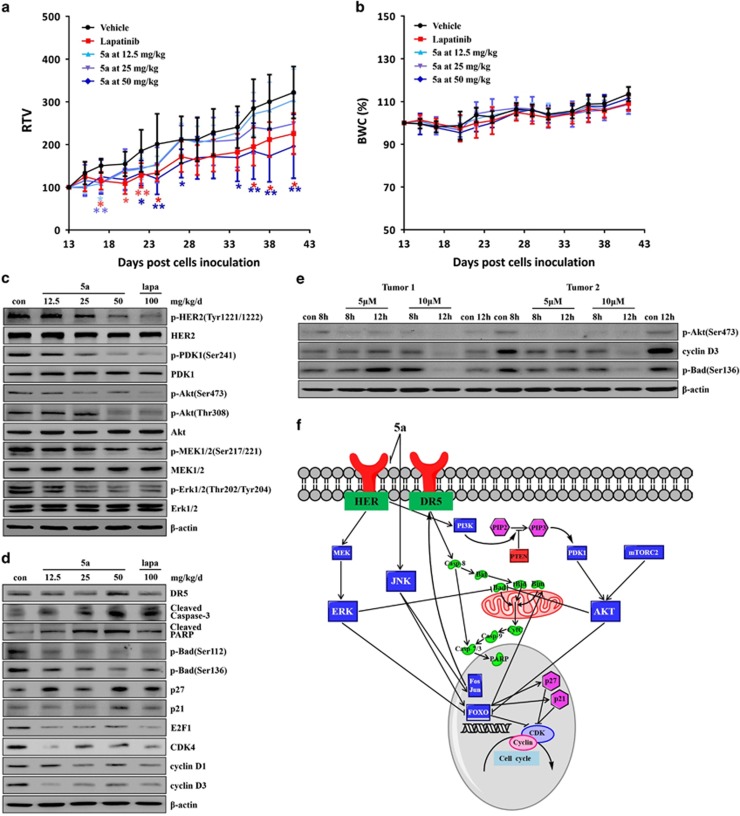
Antitumor effect of 5a *in vivo* through the inhibition of HER2 tyrosine phosphorylation and downstream PI3K/Akt and MEK/Erk pathways. (**a** and **b**) Relative tumor volume (RTV) and body weight change (%BWC) were measured as described in Materials and Methods. Statistical significance of the difference in RTV of treatment groups compared with control (*P<0.05; **P<0.01, *n*=8). (**c** and **d**) MDA-MB-453 tumor xenograft cell lysates were analyzed by immunoblotting with the antibodies indicated. (**e**) Primary breast cancer cell lines were treated with 5a at 5 or 10 *μ*M for indicated time points, cell lysates were then analyzed by western blot. (**f**) Schematic illustration of 5a-stimulated signaling networks

The data shown above prompted us to address whether the antitumor effect of 5a can work *in vivo*. Tumor growth was significantly inhibited by treatment with 5a at the highest dose level (50 mg/kg) and with lapatinib (Figure 5a). Furthermore, we did not observe any weight loss or other signs of toxicity in mice treated with 5a or lapatinib (Figure 5b).

The effects of 5a on the activation of HER2 and downstream PI3K/Akt and MEK/Erk pathways were also examined in the MDA-MB-453 xenograft. As shown in Figure 5c, 5a treatment inhibited the phosphorylation of HER2. We next examined the effects of 5a on PI3K/Akt and MEK/Erk pathways, and little or no effect was observed when mice were treated with vehicle alone or administered 12.5 mg/kg 5a. However, at 25 and 50 mg/kg doses, 5a exhibited a significant suppressive effect on the phosphorylation of PDK1, Akt, MEK and Erk1/2 in tumors (Figure 5c). Treatment with a 100 mg/kg dose of lapatinib showed the similar inhibitory effect on p-HER2 and downstream PI3K/Akt and MEK/Erk pathways as did 5a. 5A was also found to induce apoptosis and cell cycle arrest *in vivo* through the induction of DR5, p27 and p21 overexpression and activation of caspase-3 and PARP. 5A also reduced p-Bad (Ser112), p-Bad (Ser136), E2F1, CDK4 and cyclin D expression (Figure 5d). Analysis of the primary breast cancer cell lines, which were isolated from the human tumor samples, further confirmed that 5a significantly inhibited Akt Ser473 and Bad Ser136 phosphorylation and reduced cyclin D3 expression (Figure 5e). These results indicate that 5a could induce apoptosis and cell cycle arrest in breast tumors through the reduction of tyrosine phosphorylation of HER2 with subsequent inhibition of PI3K/Akt and MEK/Erk pathways *in vivo*.

## Discussion

Activation of EGFR and HER2 induces transphosphorylation of the ERBB dimer partner and stimulates intracellular pathways such as PI3K/Akt, MEK/Erk, Src kinases and activation of STAT transcription factors.^5^ The phosphorylated tyrosine 1173 of EGFR can function as a docking site for PI3K/Akt signaling systems.^33^ In addition, phosphorylated tyrosines 1173 of EGFR, 1221/1222 and 1248 of HER2 are binding sites for the adaptor proteins Shc and Grb2, which have a major role in the MEK/Erk pathway.^33, 34, 35, 36^ Thus, inhibition of phosphorylation of these tyrosines by 5a should prevent the binding of PI3K and Grb2, leading to the inhibition of activation of the PI3K/Akt and MEK/Erk pathways, causing cell cycle arrest, apoptosis and tumor regression. Therefore, the antitumor activity of 5a may act through the PI3K/Akt and MEK/Erk pathways to regulate FOXO transcription factors and the Bcl-2 family.^37, 38, 39, 40, 41^ The mammalian FOXO transcription factor family comprises four members (FOXO1, FOXO3, FOXO4 and FOXO6) that mainly differ in their tissue-specific expression. In the absence of PI3K/Akt and MEK/Erk signaling pathways, FOXO proteins, in dephosphorylated states, translocate to the nucleus, where they regulate diverse transcription targets to promote cell cycle arrest and apoptosis. In this study, we found that FOXO transcription factors blocked cell cycle progression at the G1 phase through the regulation of p27^42^ and p21.^43^

The Bcl-2, a family of structurally related molecules, has an instrumental role in the regulation of apoptosis. This family includes proapoptotic members (Bad, Bax, Bak, Puma, Bid and Bim) and antiapoptotic members (Bcl-2, Bcl-XL and Mcl-1).^44^ It has been demonstrated that members of the Bcl-2 family are critical death regulators of mitochondrial integrity. Some of them are regulated by PI3K/Akt and MEK/Erk signaling pathways through translational and posttranslational modifications. Bad binds to Bcl-XL or Bcl-2 and inhibits their antiapoptotic potential. When Bad is phosphorylated on Ser112 by Erk1/2,^45^ or Ser136 by Akt,^41, 46^ it does not exhibit proapoptotic activity in cells. Bim functions as a tumor suppressor in various cancers and contains only a protein-interaction motif known as a BH3 domain, allowing it to bind to prosurvival Bcl-2 molecules and neutralize their function.^47^ In this study, 5a inhibited the phosphorylation of Akt and Erk, leading to dephosphorylation of Bad at Ser112 and Ser136. At the same time, inactivated Akt and Erk were shown to be necessary for dephosphorylation of FOXO, which causes a Bim transcriptional downstream event to occur. Clearly, translational and posttranslational modification-dependent signal cascades are necessary for their activation and translocation to the mitochondria, leading to the release of cytochrome *c* from the mitochondria to the cytosol, and then intrinsic mitochondrial-mediated apoptosis can be initiated.

We have also found that 5a-induced cleavage and activation of caspase-8 and Bid, indicating involvement of the extrinsic DR-mediated pathway in 5a-induced apoptosis. Although it has been demonstrated that TRAIL induces the extrinsic apoptosis pathway,^29^ several cancer cell lines are resistant to the proapoptotic effects of TRAIL.^48, 49^ A large number of studies have attempted to find new anticancer agents that could augment apoptosis induced by TRAIL. The mechanism underlying the augmentation of TRAIL-induced apoptosis is mainly related to the upregulation of expression of TRAIL receptors (i.e., DR4 and DR5). Therefore, agents that upregulate the expression of DR4 and/or DR5 may have the potential for clinical management of cancer. Herein, our data have demonstrated that 5a could induce JNK phosphorylation and rapidly increase the expression of c-Jun and c-Fos, suggesting that rapid activation of the JNK pathway is induced by 5a in human breast cancer cells. This result was further confirmed using siRNAs in knockdown assays, si-JNK completely abolished 5a-induced JNK activation and upregulation of DR5. Altogether, our results emphasize the necessity of JNK activation for 5a-induced apoptosis in human breast cancer cells.

Our studies offer the first evidence of a novel 5a for its antitumor effect on human breast cancer cells. This effect was achieved through the inhibition of EGFR and HER2 activity, reducing EGFR and HER2 tyrosine phosphorylation and inhibiting downstream activation of PI3K/Akt and MEK/Erk pathways. Treatment of human breast cancer cells with 5a inhibited the phosphorylation of FOXO, promoting FOXO translocation from the cytoplasm into the nucleus. In addition, 5a activated the significant expression of DR5 through the JNK signaling pathway, resulting in a strong death response in breast cancer cells. These biochemical changes lead to cell cycle arrest at the G1 phase and induced apoptosis *in vitro* and *in vivo* (Figure 5f). In summary, we have uncovered a novel mechanism by which 5a exerts antitumor effects *in vitro* and *in vivo*. This study provides a strong proof of principle that 5a is feasible to be further developed as a novel multitarget anticancer drug for human breast cancer.

## Materials and Methods

### Cell culture

The human breast cancer cell lines, BT-474, BT-549, HCC1937, T-47D, ZR-75-1, MCF-7, MDA-MB-468, MDA-MB-453 and MDA-MB-231, were purchased from American Type Culture Collection (Manassas, VA, USA) and cultured according to the supplier's instructions.

### MTT

Cell viability was assessed by the MTT assay. Briefly, 3–8 × 10^3^ cells were seeded into 96-well plates for 12 h, followed by incubation with various doses of 5a for 72 h. After adding 10 *μ*l per well of MTT (5 mg/ml) solution, the formazan crystals were dissolved in 100 *μ*l per well DMSO. The absorbance at 490 nm was measured using Multimode Detector (Beckman Coulter, Fullerton, CA, USA). Three independent experiments were performed.

### Gene expression microarray and analyses

Human cDNA microarrays covering 35 kb cDNA spots (CapitalBio, Beijing, China) were used. In brief, MDA-MB-453 cells were exposed to vehicle or 5a for 24 h. Total RNA was extracted using Trizol (Invitrogen, Carlsbad, CA, USA), and fluorescence-labeled cDNA probes were made for hybridization using 500 ng of total RNA with T7-oligo(dT) primer and Klenow enzyme (TaKaRa, Dalian, China). Hybridized slides were scanned using a LuxScan 10 K-A confocal laser microscopy scanner (CapitalBio), and signal intensities for each spot were calculated by subtracting local background using LuxScan 3.0 software (CapitalBio). Three independent replicates were conducted, and spots with ≥2-fold increase or decrease were considered to have significant changes. Gene expression signaling pathways were analyzed with MAS3.0 software (CapitalBio). The hierarchical cluster algorithm used daverage linkage (Cluster 3.0) clustering of the genes identified by SAM with Euclidian distance and averaged linkage method.

### Cytosolic cytochrome *c* release

Mitochondria were isolated using a Cell Mitochondria Isolation Kit (Beyotime, Shanghai, China) according to the manufacturer's instructions. Cytochrome *c* release into the cytosolic fraction for each condition was assessed by western blot analysis.

### Preparation of nuclear and cytoplasmic fractions

Nuclear and cytoplasmic fractions were prepared using a Nuclear and Cytoplasmic Protein Extraction Kit (Beyotime) according to the manufacturer's instructions.

### Immunofluorescence

Cells grown on coverslips were fixed for 30 min in 4% paraformaldehyde, permeabilized and incubated in blocking buffer (3% BSA in PBS+0.5% Triton X-100) for 1 h. Cells were then incubated in dilution buffer (3% BSA in PBS+0.5% Triton X-100) containing the indicated primary antibody for 8–12 h at 4 °C and then washed extensively in PBS before being incubated with the appropriate fluorochrome-conjugated secondary antibody for 1 h. Nuclei were stained by 4′,6-diamidino-2-phenylindole.

### Animal studies

All animal studies were in accordance with and approved by the Institutional Animal Care and Use Committee of the Administrative Committee on Animal Research of the Graduate School at Shenzhen, Tsinghua University. Five-week-old female NOD/scid mice (Beijing HFK Bioscience Co. Ltd., Beijing, China) were maintained in a pathogen-free environment. Tumors were generated by transplanting 1.0 × 10^7^ MDA-MB-453 cells in a 1:1 mixture with matrigel (BD Biosciences, San Jose, CA, USA) by injection into the right flank. When the tumor volume reached an average of 100 mm^3^, the tumor bearing mice were divided into treatment groups based on tumor volume and body weight. Vehicle or 5a was administered intravenously for 7 days and then administered intraperitoneally once daily for the rest of study, whereas lapatinib was administered via oral gavage once daily. Tumor volume and body weight were measured three times per week in a blind manner. Relative tumor volume (RTV)=(TV*_n_*/TV_0_) × 100, where TV*_n_* is the TV at day *n* and TV_0_ is the TV at the day when the treatment was initiated. Percentage of body weight change (%BWC)=(BW*_n_*/BW_0_) × 100, where BW*_n_* is the BW at day *n* and BW_0_ is the BW at the day when the treatment was initiated. Data were reported as mean±S.D. To prepare lysates, tumor tissue was homogenized in lysis buffer and then processed for western blot.

### Establishment and cultivation of primary breast cancer cell lines

Primary breast cancer cell lines were obtained from human breast tumor patients whose tumors had been characterized as having HER2 overexpression. All patients gave written informed consent for their tissue samples to be used in experiments. Briefly, freshly isolated tumor tissues were minced with sterile razor blades, digested with collagenase I, collagenase II, collagenase III, collagenase IV and hyaluronidase (1 mg/ml each) for 2–3 h at 37 °C. The cells were resuspended in DMEM/F12 (Gibco, Carlsbad, CA, USA) supplemented with 10% FBS (Gibco) and the cells were strained through a 40 *μ*m sieve. Cells were harvested and seeded into 60 mm dishes in DMEM/F12 supplemented with 20% FBS. Cells were passaged by trypsinization. All studies were carried out on cells cultivated for fewer than five passages.

## Figures and Tables

**Table 1:** The IC_50_ of 5a and lapatinib on various breast cells and the corresponding levels of EGFR and HER2 expression as measured by western blot

**Cell line**	**EGFR**	**HER2**	**IC**_**50**_ **(μM) (5a)**	**IC**_**50**_ **(μM) (lapatinib)**
MDA-MB-468	++++	−	3.31±0.30	6.06±0.41
BT-549	++	−	3.01±0.08	8.48±1.55
MDA-MB-231	+	−	3.73±0.37	5.20±0.10
HCC1937	+	−	9.02±1.26	2.97±0.10
T-47D	+	+	7.89±0.28	3.47±0.12
BT-474	−	++++	3.58±0.10	0.038±0.003
MDA-MB-453	−	++	4.91±0.44	0.19±0.098
ZR-75-1	−	+	1.81±0.14	6.26±1.90
MCF-7	−	−	2.99±0.60	5.60±0.76
MCF-10 A	+++	+	17.33±3.14	24.74±5.63

**++++** means very strong expression of EGFR or HER2; **+++** means strong expression of EGFR or HER2; **++** means moderate expression of EGFR or HER2; **+** means low expression of EGFR or HER2; **−** means no expression of EGFR or HER2

## Abbreviations

EGFR: epidermal growth factor receptor
HER2: human epidermal growth factor receptor 2
JNK: c-Jun N-terminal kinase
DR4: death receptor 4
DR5: death receptor 5
RTK: receptor tyrosine kinase
HER3: human epidermal growth factor receptor 3
HER4: human epidermal growth factor receptor 4
TNF: tumor necrosis factor
TRAIL: TNF-related apoptosis-inducing ligand
PARP-1: poly (ADP-ribose) polymerase-1
CDK: cyclin-dependent kinase
PARP: poly (ADP-ribose) polymerase
siRNA: small interfering RNA
