# Supplementary material for: A benzimidazole derivative exhibiting antitumor activity blocks EGFR and HER2 activity and upregulates DR5 in breast cancer cells
Source: Cell Death Dis. 2015 Mar 12;6(3):e1686–. doi: 10.1038/cddis.2015.25 (PMC4385914; doi:10.1038/cddis.2015.25)
Supplement: Supplementary Information [file cddis201525x1.doc]

**A benzimidazole derivative exhibiting antitumor activity blocks EGFR and HER2 activity and upregulates DR5 in breast cancer cells.**

Bizhu Chu^1,2^, Feng Liu^2,3,*^, Lulu Li^2^, Chao Ding^1,2^, Kang Chen^1,2^, Qinsheng Sun^2^, Zhifa Shen^4^, Ying Tan^2,3^, Chunyan Tan^2,3^, and Yuyang Jiang^5,2,3,*^

**Supplementary data**

**Supplemental materials and methods**

**Reagents and antibodies**

phospho-EGFR (Tyr1173), EGFR, phospho-HER2 (Tyr1221/1222), phospho-HER2 (Tyr1248), HER2, phospho-PDK1 (Ser241), PDK1, phospho-Akt (Ser473), phospho-Akt (Thr308), Akt, phospho-MEK1/2 (Ser217/221), MEK1/2, phospho-p44/42 MEK (Erk1/2) (Thr202/Tyr204), p44/42 MEK (Erk1/2), phospho-FOXO1 (Ser319), phospho-FOXO1 (Ser256), phospho-FOXO1 (Thr24)/FOXO3a (Thr32), FOXO1, FOXO3a, phospho-SAPK/JNK (Thr183/Tyr185), SAPK/JNK, phospho-Bad (Ser112), phospho-Bad (Ser136), Bad, Bim, Bid, Cyclin D1, Cyclin D2, Cyclin D3, p27 Kip1, p21 Waf1/Cip1, p18 INK4C, p15 INK4B, CDK2, CDK4, Cleaved Caspase-9, Cleaved Caspase-8, Cleaved Caspase-7, Cleaved Caspase-3, Cleaved PARP, PARP, Cytochrome c, Cox IV, β-actin, Anti-rabbit IgG, Anti-mouse IgG and F(ab')2 Fragment (Alexa Fluor® 488 Conjugate) were obtained from Cell Signaling Technology. E2F1 and Bcl-2 were purchased from Santa Cruz Biotechnology. CDK1 was purchased from BD Biosciences. Bcl-XL was obtained from Bioworld Technology. DR5 was obtained from Abcam. Collagenase I, collagenase II, collagenase III, collagenase IV and hyaluronidase were purchased from Sigma-Aldrich. Lapatinib was obtained from Selleck Chemicals.

**Colony formation**

0.1-1 × 10^3^ of cells were seeded into six-well plates and cultured for 12 h. Then the medium was replaced by 5a at the indicated dilutions. After being incubated for another 14 or 20 days, colony formed by breast cancer cells were fixed with 100% cold ethanol and then stained with 0.05% crystal violet for 30 min.

**Morphological analysis**

Cells were plated and exposed to vehicle (1% DMSO) or various concentrations of 5a for 24-36 h. Cells were then fixed with 4% paraformaldehyde for 10 min. Fixed cells were washed with PBS, incubated with Hoechst-33258 (10 mg/mL, Sigma) for 10 min in dark at room temperature, cells were washed three times with PBS. Images were taken, using a camera Olympus fluorescent microscope with 330 to 380 nm excitation filter.

**Apoptosis assay**

Apoptosis measurement with flow cytometry was carried out using Alexa Fluor® 488 Annexin V/Dead Cell Apoptosis Kit (Invitrogen). Briefly, cells were plated in 60 mm dishes and the following day cells were exposed to vehicle or 5a for the indicated times, both adherent and floating cells were harvested, washed twice with ice cold PBS, resuspended in 100 µL incubation buffer containing annexin V-FITC and PI, incubated in the dark for 15 min and analyzed by fluorescence activated cell sorting (FACS).

**Cell cycle analysis**

Cells were plated in 60 mm dishes and treated with vehicle or 5a at the concentrations indicated for 24 h. Cells were then trypsinized, collected and washed in PBS. Cells were subsequently fixed in 70% ethanol, stored overnight at -20 °C, washed and stained with PI staining reagent (20 µg/mL PI, 200 µg /mL RNase, 0.1% Triton-X 100) and incubated in the dark for 30 min. The percentage of cells in each phase of the cell cycle was measured by FACS.

**Preparation of total protein**

After washing the cell twice in PBS, whole cell extracts were prepared by scraping cells off petri dishes in ice cold lysis buffer [10 mM HEPES (pH 7.9), 10 mM KCl, 1 mM EDTA, 0.1% NP-40] containing freshly added protease and phosphatase inhibitors cocktails (Roche) and incubated for 30 min on ice. Lysates were cleared by centrifugation at 20000 g, 4 °C for 10 min and the supernatant was snap frozen in -80 °C.

**Western blot**

20-40 μg protein was separated by 7.5%-15% SDS-PAGE and transferred to PVDF membrane (Roche). Membranes were blocked for 2 h in 1 × TBST containing 5% (w/v) BSA, and then incubated with a primary specific antibody in 5% of BSA for 8-12h at 4 °C. Followed by a HRP-conjugated anti-mouse or anti-rabbit second antibodies, proteins were visualized with the SuperSignal West Pico Chemiluminescent Substrate kit (Pierce).

**RNA interference**

For RNA interference, cells were seeded at a density of 2 × 10^5^/well in 60 mm dishes. Transfection of 75 nM siRNA was carried out using Lipofectamine 2000 (Invitrogen). For all procedures, standard protocols were used according to the manufacturer’s instructions.

Synthetic siRNAs targeting HER2 and JNK were purchased from GenePharma. Target sequences and nonspecific siRNA (NC) sequences were as follows:

| Gene Name | Sense (5’-3’) | Antisense (5’-3’) |
| --- | --- | --- |
| HER2 #1 | GGGGCUGGCUCCGAUGUAUtt | AUACAUCGGAGCCAGCCCCtt |
| HER2 #2 | UCUCUGCGGUGGUUGGCAUUC | AUGCCAACCACCGCAGAGACG |
| JNK1 #1 | GGCAUGGGCUACAAAGAAAtt | UUUCUUUGUAGCCCAUGCCtt |
| JNK1 #2 | AAGCCCAGUAAUAUAGUAGUAtt | UACUACUAUAUUACUGGGCUUtt |
| JNK2 #1 | GACUCAACCUUCACUGUCCUAtt | UAGGACAGUGAAGGUUGAGUCtt |
| JNK2 #2 | CAUGAUGUUAUCAUAUCUUAUtt | AUAAGAUAUGAUAACAUCAUGtt |
| NC | UUCUCCGAACGUGUCACGUtt | ACGUGACACGUUCGGAGAAtt |

**RNA isolation and real-time PCR analysis**

Total RNA was extracted using Trizol (Invitrogen) following the manufacturer’s instructions. cDNA synthesis was performed with 400 ng of total RNA using the PrimeScript™ RT Master Mix (Perfect Real Time) kit (TaKaRa). Gene expression was quantified with SYBR® Premix Ex Taq™ (Tli RNaseH Plus) kit (TaKaRa) on the ABI 7500 (Applied Biosystems). Results were normalized to GAPDH and analyzed using SDS 1.4 software.

The following primers were used in real-time PCR for measuring gene expression relative to GAPDH.

| Gene | Forward (5’-3’) | Reverse (5’-3’) |
| --- | --- | --- |
| GAPDH | TGGCAAATTCCATGGCACCG | CGCCCCACTTGATTTTGGAGG |
| DR4 | GCAGCTGGACCTCACGAAAA | CCTGGGCCTGCTGTACCA |
| DR5 | GGCCACAGGGACACCTTGTA | TCGCCCGGTTTTGTTGA |
| c-Fos | GAGGGGCAAGGTGGAACAGT | CTTGCAGGCAGGTCGGTGAG |
| c-Jun | TGCCCCAAGAACGTGACAGAT | GCCTGGGTTGAAGTTGCTGAG |

**Supplementary figures**


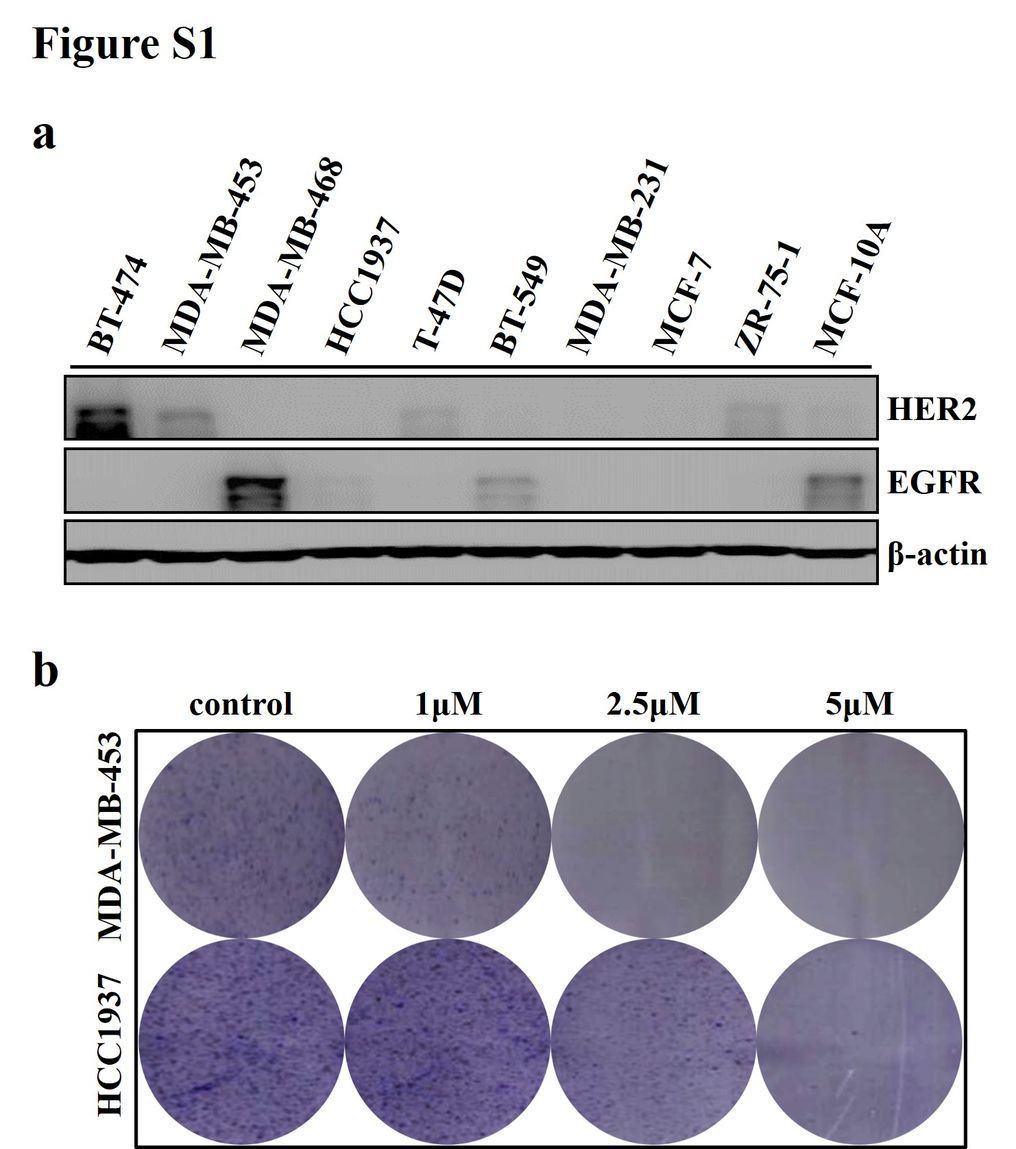


Figure S1 (a) Expression of EGFR and HER2 were analyzed in breast cancer cell lines. Cell lysates from the various breast cancer cell lines were probed for EGFR and HER2 content. (b) Cell viability, as determined by colony formation assay, was assessed in breast cancer cells treated with 5a.


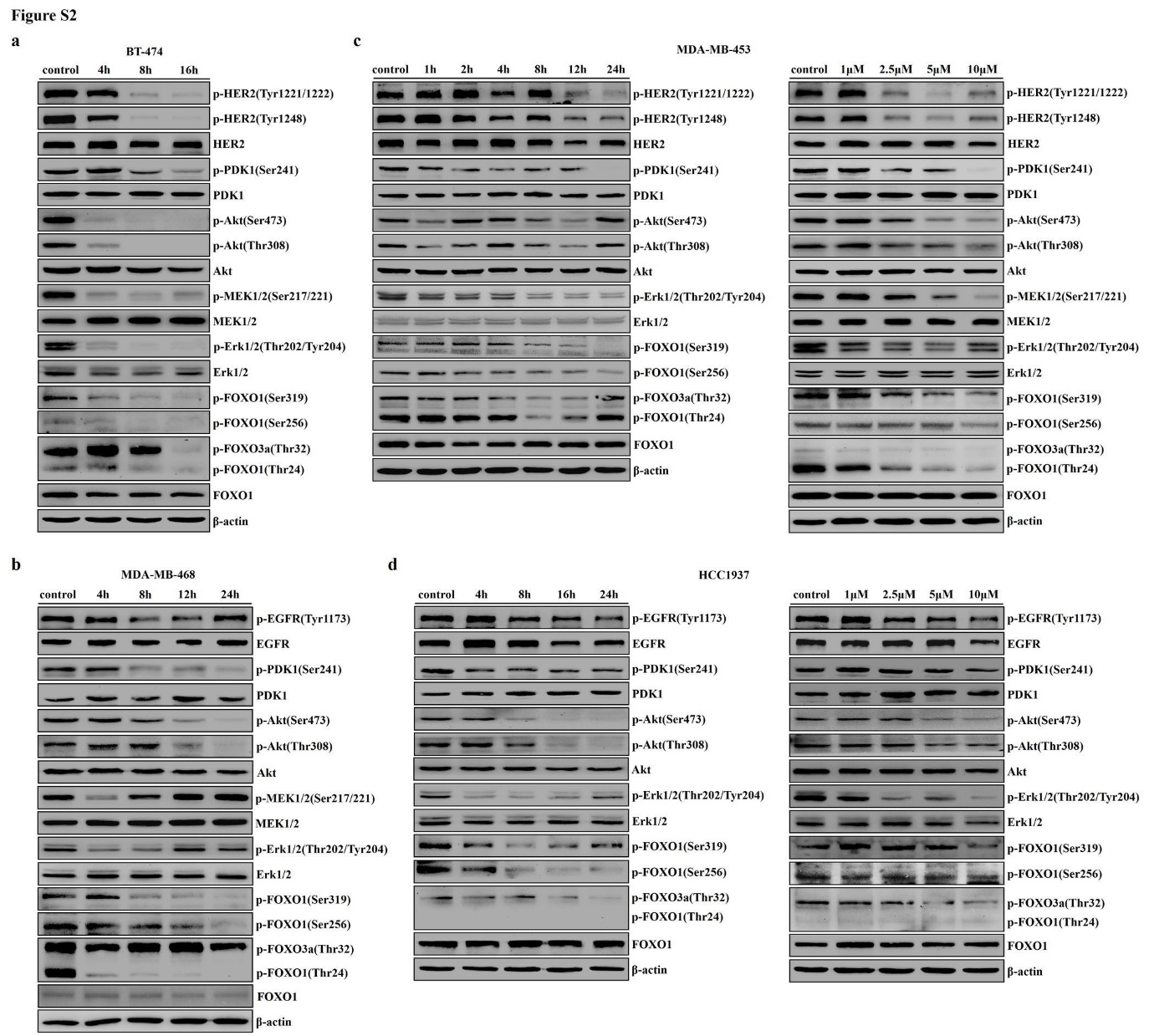


Figure S2 5a inhibited EGFR/HER2 tyrosine phosphorylation and downstream signaling pathways. (a-b) BT-474 and MDA-MB-468 cells were treated with 5a at 10 μM for indicated time points, cell lysates were analyzed by immunoblotting with the antibodies indicated. (c) MDA-MB-453 cells were treated with 5a at 5 μM for indicated time points or treated with 5a for 12 h with different concentrations as indicated, cell lysates were analyzed by immunoblotting with the antibodies indicated. (d) HCC1937 cells were treated with 5a at 10 μM for indicated time points or treated with 5a for 16 h with different concentrations as indicated, cell lysates were analyzed by immunoblotting with the antibodies indicated.


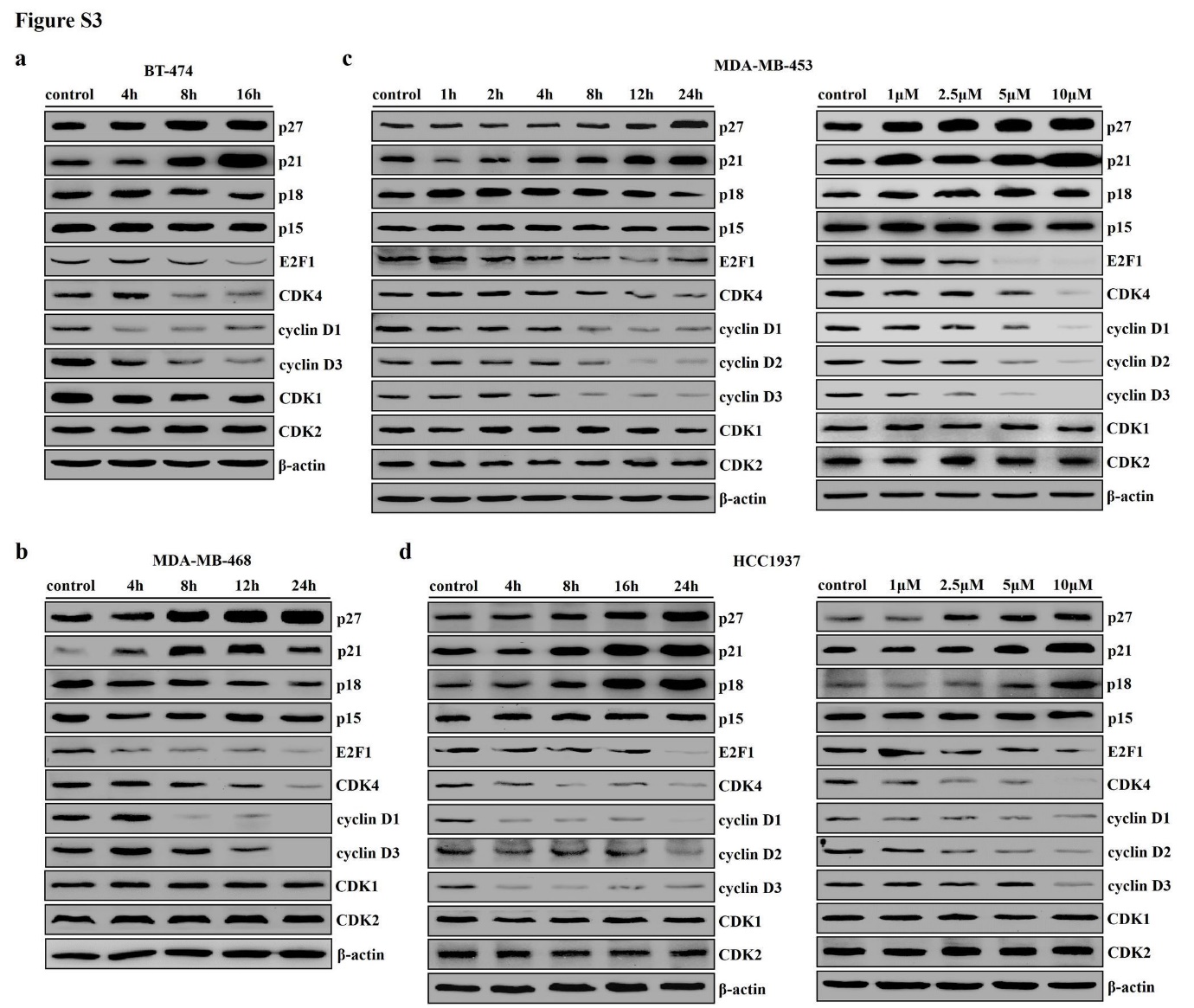


Figure S3 5a causes G1 arrest in breast cancer cells. (a-b) BT-474 and MDA-MB-468 cells were treated with 5a at 10 μM for indicated time points, cell lysates were analyzed by immunoblotting with the antibodies indicated. (c) MDA-MB-453 cells were treated with 5a at 5 μM for indicated time points or treated with 5a for 12 h with different concentrations as indicated, cell lysates were analyzed by immunoblotting with the antibodies indicated. (d) HCC1937 cells were treated with 5a at 10 μM for indicated time points or treated with 5a for 16 h with different concentrations as indicated, cell lysates were analyzed by immunoblotting with the antibodies indicated.


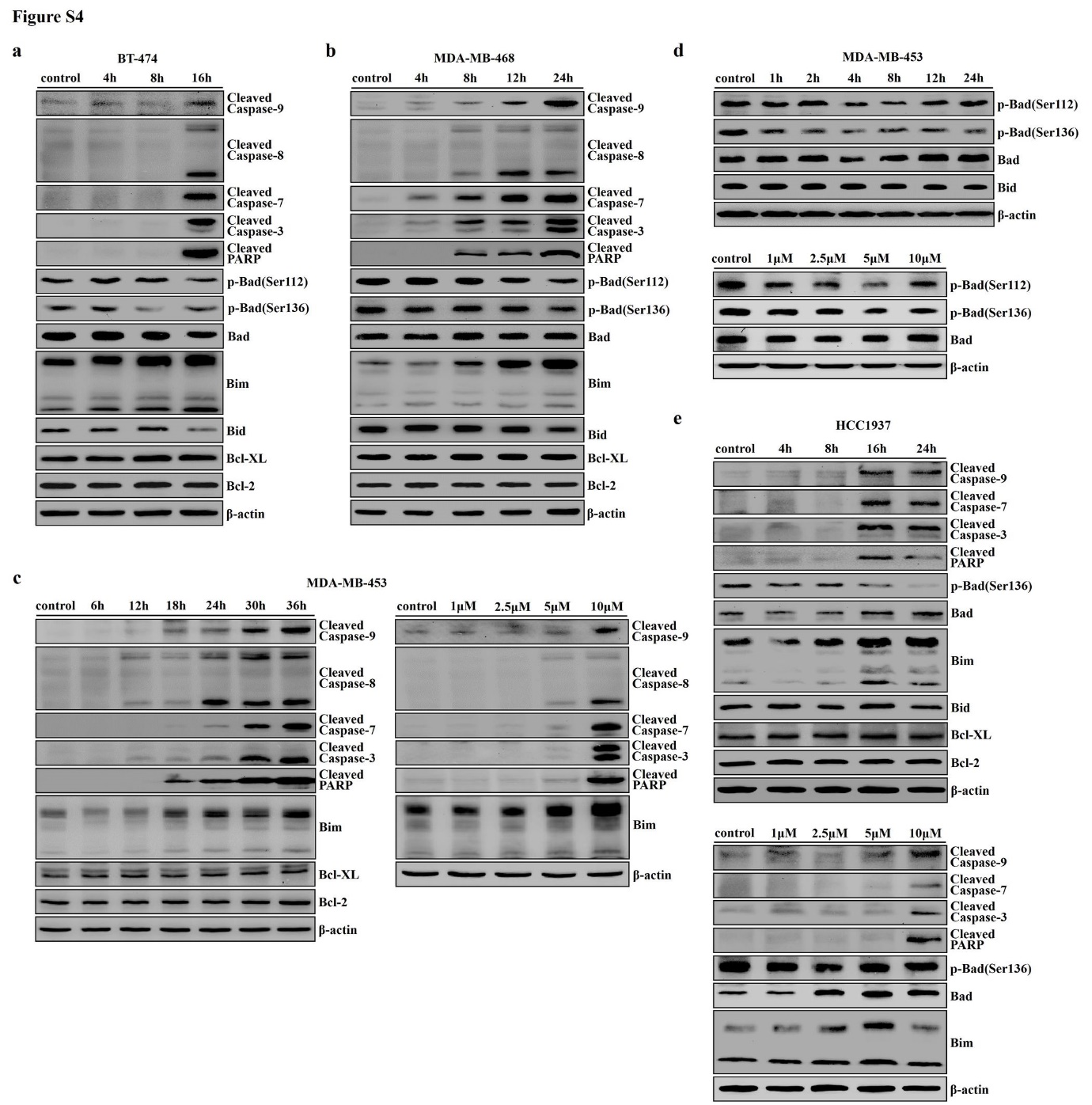


Figure S4 5a induced apoptosis in breast cancer cells. (a-b) BT-474 and MDA-MB-468 cells were treated with 5a at 10 μM for indicated time points, cell lysates were analyzed by immunoblotting with the antibodies indicated. (c) MDA-MB-453 cells were treated with 5a at 10 μM for indicated time points or treated with 5a for 36 h with different concentrations as indicated, cell lysates were analyzed by immunoblotting with the antibodies indicated. (d) MDA-MB-453 cells were treated with 5a at 5 μM for indicated time points or treated with 5a for 12 h with different concentrations as indicated, cell lysates were analyzed by immunoblotting with the antibodies indicated. (e) HCC1937 cells were treated with 5a at 10 μM for indicated time points or treated with 5a for 16 h with different concentrations as indicated, cell lysates were analyzed by immunoblotting with the antibodies indicated.


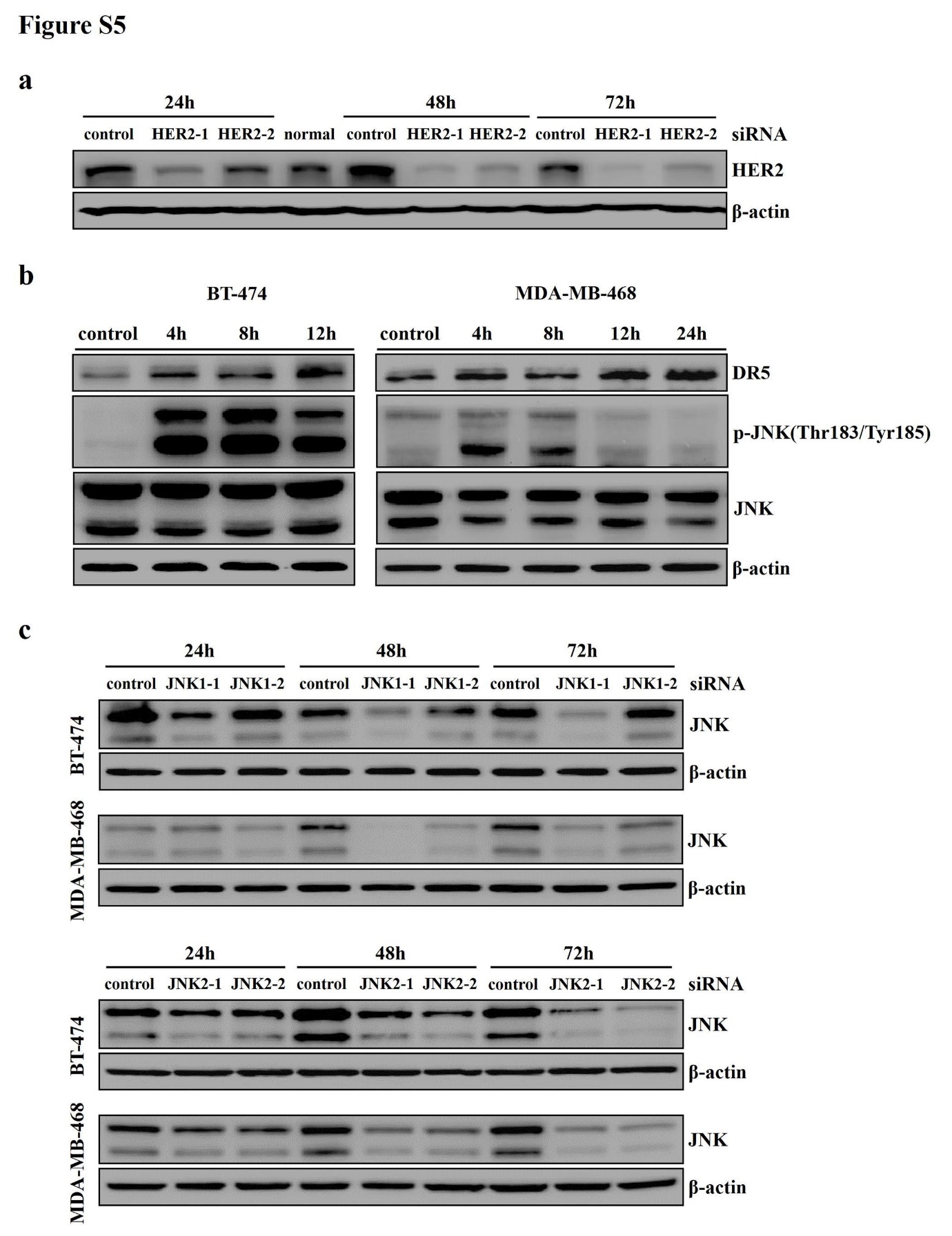


Figure S5 (a) BT-474 cells were transfected with control or HER2 siRNA for 24 h, 48 h and 72 h. Cell lysates were analyzed by immunoblotting with the antibodies indicated. (b) BT-474 and MDA-MB-468 cells were treated with 5a at 10 μM for indicated time points, cell lysates were analyzed by immunoblotting with the antibodies indicated. (c) BT-474 and MDA-MB-468 cells were transfected with control or JNK siRNA for 24 h, 48 h and 72 h. Cell lysates were analyzed by immunoblotting with the antibodies indicated.
